# Supplementary material for: AI in dermatology: a comprehensive review into skin cancer detection
Source: PeerJ Comput Sci. 2024 Dec 5;10:e2530. doi: 10.7717/peerj-cs.2530 (PMC11784784; doi:10.7717/peerj-cs.2530)
Supplement: Supplemental Information 1 [file peerj-cs-10-2530-s001.docx]

Annexure 1 Categorization of literature based on a classification framework

|  | **Authors** | **Category & Type** | **Observations** |
| --- | --- | --- | --- |
| **Advantages** | Kaplan & Haenlein (2020), Farhud & Zokaei (2021), Carayon et al. (2018), Pee et al. (2018), Gandomi et al. (2022), Tobore et al. (2019), Khater et al. (2023), Priyadharshini et al. (2023), Kumar K et al. (2023), Abbas et al. (2023), Kumar et al. (2022), Meena & Hasija (2022), Patel et al. (2021), Hossain et al. (2020), Hekler et al. (2019), Yehia et al. (2019), Jin et al. (2019), Mobiny et al. (2019 | Medical Sector | **Database sharing:** Early diagnosis, Process ease, Patient monitoring, Automated decision making.  **Professional Training:** Organizational workflow assistance, Improved performance, Professional training, Data sharing, and availability.  **Resource Utilization:** The medical sector consumes fewer resources, provides basic training, saves time, and has data availability. - **Insurance and Claim management:** Improved management and efficiency in handling claims and insurance processes. |
|  | Carayon et al. (2018), Pee et al. (2018), Gandomi et al. (2022), Khater et al. (2023), Maqsood & Damaševičius (2023), Shahin et al. (2023), Kumar K et al. (2023), Magdy et al. (2023), Abbas et al. (2023), Kumar et al. (2022), Meena & Hasija (2022), Patel et al. (2021), Carvalho et al. (2021), Hossain et al. (2020), Yehia et al. (2019) | Clinical Specific/Organizations | **Work order management, KPIs:** Organizational workflow assistance, Improved performance, Professional training, Data sharing, and availability.  **Cost-saving models:** Improved efficiency and reduced costs in clinical settings. |
|  | Kaplan & Haenlein (2020), Farhud & Zokaei (2021), Sturmberg & Bircher (2019), Strachna & Asan (2020), Istepanian & Al-Anzi (2018), Priyadharshini et al. (2023), Mridha et al. (2023), Abbas et al. (2023), Kumar et al. (2022), Biasi et al. (2022), Patel et al. (2021), Hossain et al. (2020), Hekler et al. (2019), Yehia et al. (2019), Jin et al. (2019), Mobiny et al. (2019) | Patient | **Automated decision-making:** Enhanced decision-making processes for patient treatment and monitoring.  **Patient treatment & monitoring:** Simplified Process & Early Detection, Increased efficiency in terms of cost & time.  **Simplified Process & Early Detection:** Improved early detection of conditions, leading to better patient outcomes.  **Increased efficiency in cost & time:** Reduction in costs and time required for treatment and monitoring. |
| **Challenges** | Kaplan & Haenlein (2020), Carayon et al. (2018), Pee et al. (2018), Istepanian & Al-Anzi (2018), Gandomi et al. (2022), Tobore et al. (2019), Khater et al. (2023), Mbunge & Batani (2023), Maqsood & Damaševičius (2023), Shahin et al. (2023), Kumar K et al. (2023), Meena & Hasija (2022), Moloud Abdar et al. (2021), Patel et al. (2021), Hossain et al. (2020), Hekler et al. (2019), Yehia et al. (2019), Jin et al. (2019), Mobiny et al. (2019) | Safety of the patient | **Privacy, Legal, and Compliance with Government Regulations Issues**: Concerns about patient privacy, legal implications, and compliance with government regulations when using AI in healthcare. |
|  | Carayon et al. (2018), Pee et al. (2018), Istepanian & Al-Anzi (2018), Gandomi et al. (2022), Tobore et al. (2019), Khater et al. (2023), Mbunge & Batani (2023), Maqsood & Damaševičius (2023), Shahin et al. (2023), Kumar K et al. (2023), Meena & Hasija (2022), Moloud Abdar et al. (2021), Patel et al. (2021), Hossain et al. (2020), Hekler et al. (2019), Yehia et al. (2019), Jin et al. (2019), Mobiny et al. (2019) | Human Interventions | **Data availability, digitalization, and consolidation**: Challenges related to data availability, digitization, and consolidation for AI applications.  **Patient treatment errors, data errors, decision errors, and human intervention**: Potential for errors in patient treatment, data processing, and decision-making due to human intervention. |
|  | Kaplan & Haenlein (2020), Carayon et al. (2018), Lohachab et al. (2020), Pee et al. (2018), Istepanian & Al-Anzi (2018), Gandomi et al. (2022), Tobore et al. (2019), Khater et al. (2023), Mbunge & Batani (2023), Maqsood & Damaševičius (2023), Shahin et al. (2023), Kumar K et al. (2023), Munappy et al. (2022), Meena & Hasija (2022), Moloud Abdar et al. (2021), Patel et al. (2021), Jiang et al. (2021), González-Cruz et al. (2020), Hekler et al. (2019), Yehia et al. (2019), Jin et al. (2019), Mobiny et al. (2019) | Legal Issues and Privacy | **Legal and Privacy issues:** Concerns about the legal implications and privacy issues related to the use of AI in healthcare.  **Compliance with Govt regulations:** Need to comply with government regulations regarding the use of AI in medical applications. |
|  | Istepanian & Al-Anzi (2018), Gandomi et al. (2022), Tobore et al. (2019), Hwang et al. (2016), Khater et al. (2023), Mbunge & Batani (2023), Maqsood & Damaševičius (2023), Shahin et al. (2023), Kumar K et al. (2023), Munappy et al. (2022), Meena & Hasija (2022), Moloud Abdar et al. (2021), Patel et al. (2021), Jiang et al. (2021), González-Cruz et al. (2020), Hekler et al. (2019), Yehia et al. (2019), Jin et al. (2019), Mobiny et al. (2019) | Database Integration | **Database Availability**: Challenges in ensuring the availability of comprehensive and accurate databases for AI training.  **Database consolidation**: Issues related to consolidating databases from different sources for effective AI application. |
| **Methodologies** | Zhou et al. (2017), Shinde et al. (2022), Kanchana Sethanan et al. (2023), Priyadharshini et al. (2023), Çağrı Suiçmez et al. (2023), Venugopal et al. (2023), Kumar K et al. (2023), Mridha et al. (2023), Gururaj et al. (2023), Magdy et al. (2023), Lee & You (2023), Zhou et al. (2023), Manar Elshahawy et al. (2023), Ogundokun et al. (2023) | Database Processing | **Machine Learning and Data processing**: Techniques and methods for processing and analyzing large datasets using machine learning algorithms. **Expert Systems**: Use of AI-based expert systems for decision-making and diagnostics. |
|  | Rahman et al. (2020), Weng & Zhu (2021), Yousef Alsahafi et al. (2023), Wan et al. (2023), Zhou et al. (2017), Shinde et al. (2022), Mridha et al. (2023), Gururaj et al. (2023), Magdy et al. (2023), Lee & You (2023), Zhou et al. (2023), Manar Elshahawy et al. (2023), Ogundokun et al. (2023) | Multimedia Processing | **Image and Video Processing, Virtual Reality**: Application of AI in processing medical images and videos and virtual reality technologies for enhanced diagnostics and treatment. |
| **Functionality** | Khan et al. (2022), Gershenwald et al. (2017), Combalia et al. (2022), Meena & Hasija (2022), Carvalho et al. (2021), Hekler et al. (2019) | Medical Sector | **Medical Imaging:** Utilization of AI to enhance medical imaging techniques.  **Database Collection Research:** Efficient data collection and research methodologies to improve medical data handling. |
|  | Kumar K et al. (2023), Mridha et al. (2023), Ogundokun et al. (2023), Behara et al. (2023), Frederico & Krohling (2022), Combalia et al. (2022), Kumar et al. (2022), Han et al. (2022), | Patient | **Consultancy Research, Diagnosis, Procedure Monitoring**: AI's role in patient consultation, diagnosis, and monitoring. |
|  | Mbunge & Batani (2023), Kanchana Sethanan et al. (2023), Maqsood & Damaševičius (2023), Shahin et al. (2023), Kumar K et al. (2023), Mridha et al. (2023), Ogundokun et al. (2023), Behara et al. (2023), Frederico & Krohling (2022), Combalia et al. (2022) | Clinic Specific | **Decision-Making and Information Sharing**: Enhancing clinical decision-making processes and information sharing among clinics. |
